# Alcohol: Its Use and Abuse

**Published:** 1874-08

**Authors:** W. L. Lipscomb

**Affiliations:** Columbus, Miss.


Vol. IV.—AUGUST, 1874.—No. 8.

### Original Communications.

### ALCOHOL: ITS USE AND ABUSE.

BY W. L. LIPSCOMB, M. D., OF COLUMBUS, MISS.

It is needless for your Essayist to state to this intelligent Association, that great diversity of opinion exists as to the action of alcohol upon the human system.

This has always been the case, and will be so to the end of time. It cannot be otherwise, while men's minds are different—it could not be different, unless the knowledge of man approached the infinitude of God.

Alcohol represents one of those great agencies in the natural universe, that touches so directly upon the function of *vitality,* that the Supreme Architect has purposely removed it from the perfect comprehension of the human mind—or the human mind has become so clouded by the effects of *sin,* that it is unable to grasp it.

Life, viewed from its great dual stand point of construction and destruction, of organization and disorganization, of growth and decay, finds in alcohol one of those important supplementary agencies whose claims cannot be disregarded, and whose *use* will be continued (despite terrible evils resulting from its *abuse*) as long as man aspires to perfection of function and duration of life on the one hand, and battles against weakness and death on the other.

Take the circle of human existence and place life at any given point, and you find alcohol, one of those great incomprehensible agencies, that stands ready with appropriate balances to preserve it, in its appointed place, and to restore it in the event of dislocation.

The dream of the Alchemist failed, because of a want of a complete knowledge of the use of alcohol, as applied to the diseases of man. The distinguished author of the Brunonian System, with one hand throttling the monster disease, and holding his botttle of brandy in the other, presented no caricature of the true physician. The only error in the picture was the dimness of his intellectual eye. He could not bring the power of the one against the evil of the other.

What is Life? Life is the blood. Nutrition is the dominant function of existence. Disease is a disorder of nutrition.

Life is electricity, with the brain for its battery and the nerves for its wires of distribution. Disease is a disorder of innervation.

Life is heat. Death is cold. Disease is a disorder of temperature.

As a presiding angel over the great fountains of life, or, if you prefer it, as a vigilant sentinel guarding the doors of the *atria mortis*, stands alcohol.

Has nutrition become feeble from the want of a requisite supply of food? Alcohol supplements the deficiency, by arresting the waste of time, until the supply can be increased. Has nutrition become deranged by the presence of inappropriate material? Alcohol supplements the forces of digestion and overcomes the resistance. Is nutrition failing because of a tardiness in the elements, to reach their appropriate destination? Alcohol sends the blood faster to its place of duty. Is nutrition heavy with excess of effete matter? Alcohol can put the heart and lungs and every emunctory on double duty to effect its discharge. Does nutrition need assistance, in the assimilation of the selected elements? Alcohol claims especial control in that department.

But disease is a disorder of innervation! Alcohol refreshes the weary brain and stimulates the exhausted nerves. Alcohol obtends sensitiveness and deadens pain. Alcohol brings refreshing sleep and averts the blow of the deadly poison. Alcohol supplies mental deficiency and controls the excess of the maniac.

But life is heat! Disease is a disorder of temperature! Alcohol is the friend of heat, and a most active anti-pyretic. Alcohol warms if you are cold, and cools if you are hot. It is alike the enemy of *shock* and the foe of *fever*. It puts the thermometer at exactly 98° and keeps it there, despite lazy lungs and an unfaithful stomach. Alcohol strides the whole domain of temperature, and assists or controls the supply of animal heat, at any or all of its numerous sources.

If alcohol has these influences upon the centres and functions of life, is it a wonder that there should exist in all nations and among all people a natural appetite and a ready acquisition of its habitual use? Is it a wonder that it has become the poor man's friend and the rich man's delight? The lethean of the miserable and the catholicon of the sick?

Will any intelligent physician contradict a single item in the lists of benefits we have ascribed to it? They are the result of positive and repeated experiments. The records of medicine re f ull of their repetition, and the great outside world knows their truth by general observation.

Alcohol, taking into consideration the breadth of its range, the extent of its power, the ease of its application, the universality and cheapness of its production, is the *chiefest remedy* for the ills of life; the most efficient agent in the cure and prevention of diseases; the grand, supplementary, medical desideratum of man's physical and mental being, in its present imperfect condition, and certain termination in decay and death.

But an agency so powerful for *good* is equally powerful for *evil*.

Force power, in any department of nature misapplied, produces disorder, friction and destruction. So alcohol is a force, a mighty force; a power, a tremendous power; and whenever applied is either *good* or *evil*. No matter what the motive or intention dictating its use, its laws are inexorable—it will benefit or injure—it will kill or cure.

The draught of ignorance, the cup of science, nor the hand of benevolence, can restrain its inherent activity or power.

The world is full of the evils of its application; disease fattens on its ignorant use; death and hell luxuriate on its abused potentiality; men see the evil everywhere; the temperate proclaim from every pulpit and rostrum; the poor inebriate does not deny it; medicine, intelligent medicine, looks with horror and dismay at its frightful ravages; more evil results to man, from alcohol, than all other agencies put together; it engenders disease in every part of man's organism; it interferes with every function of life; it accelerates all the forces of decay and death.

And the evil is constantly increasing; the bad effects are accumulating in terrible ratio's; its power and influences are irresistable, and threaten the whole world with misery and ruin.

What a wonderful combination for good and evil in a single agency! What a contradiction of results! What a problem for study! What a field of investigation for the philosopher, the scientist and the Christian.

Any attempt to enter this arena of thought, would require more time than your Essayist feels permitted to use; but he asks permission to present the following propositions:

1. The extensive and powerful agency of alcohol for *good* to the human organism, is a demonstrated and a demonstrable fact.

2. The *evil* effects are as much more extensive and powerful, as *sin* exceeds *virtue* and *ignorance* exceeds *knowledge.*

3. That the *good* may be enjoyed and the *evil* avoided, by the establishment of virtue and knowledge, and the eradication of sin and ignorance.

4. That Christianity and education are the only known factors of this desirable result.

To arrest the spread of this terrific evil, until these two forces can be properly developed and operated, your Essayist would recommend that mankind be taught by every conceivable method, the following truths, regarding alcohol:

1. That alcohol is a *poison,* and not an aliment or food; and should never, in any case, be taken by a person in health.

2. That the *poisonous* effects of alcohol are discoverable in a desire or thirst for its use; and whether that thirst or use be temperate or intemperate, a certain and progressive poisonous effect is going on in the system.

3. That the poisonous effects of alcohol are transmissable from parent to offspring.

4. That the benefits of alcohol in *disease* are limited by a return to health, and that its poisonous effects are *antidoted* by the disease it cures. Hence, alcohol properly administered in disease, never produces drunkenness.

5. That alcohol is much more dangerous taken in health than in disease.

6. That the manufacture of alcohol, *like that of money*, should be the province and duty of the government, and under its exclusive jurisdiction.

7. That the improper use and sale of alcohol should be put on a footing with other *poisons* and the offenders punished by law.

8. That the medical profession owe to themselves, to science, and the great cause of Christianity and education, a demonstration of the benefits of the proper use of alcohol, and a plain, faithful, scientific exposition of the evils resulting from its abuse.

Read before the Columbus and Lowndes County (Miss.) Medical Society, May, 1874, and requested for publication.

A. A. Lyon, M.D., President.

F. W. Spillman, M.D., Secretary.

